# Dissemination of high-level mupirocin-resistant CC22-MRSA-IV in Saxony

**DOI:** 10.3205/dgkh000304

**Published:** 2017-11-20

**Authors:** Stefan Monecke, Antje Ruppelt-Lorz, Elke Müller, Annett Reissig, Alexander Thürmer, Anna C. Shore, David C. Coleman, Ralf Ehricht, Lutz Jatzwauk

**Affiliations:** 1 Alere Technologies GmbH (Abbott Rapid Diagnostics), Jena, Germany; 2 Institute for Medical Microbiology and Hygiene, Faculty of Medicine “Carl Gustav Carus”, Technische Universität Dresden, Germany; 3 InfectoGnostics Research Campus Jena, Jena, Germany; 4 Microbiology Research Unit, Dublin Dental University Hospital, University of Dublin, Trinity College Dublin, Ireland; 5 Department of Hospital Infection Control, University Hospital, Dresden, Germany

**Keywords:** methicillin-resistant Staphylococcus aureus, MRSA, mupirocin, high-level mupirocin resistance, mupA, “Barnim Epidemic Strain”

## Abstract

Mupirocin is used for eradicating methicillin-resistant *S. aureus* (MRSA) in nasal colonization. A plasmid-borne gene, *mupA*, is associated with high-level mupirocin resistance. Despite the fact that, among all MRSA from a tertiary care center in the German state of Saxony, the prevalence of *mupA*, encoding high-level mupirocin resistance, was approximately 1% over a 15-year period from 2000–2015, a sharp increase to nearly 20% was observed in 2016/2017. DNA microarray profiling revealed that this was due to the dissemination of a variant of CC22-MRSA-IV (“Barnim Epidemic Strain” or “UK-EMRSA-15”), which, in addition to *mecA*, harbors *mupA*, *aacA-aphD*, *qacA*, and – in most isolates – *erm*(C). In order to prevent therapy failures and a further spread of this strain, the use of mupirocin should be more stringently controlled as well as guided by susceptibility testing. In addition, MRSA decolonization regimens that rely on other substances, such as betaisodona, polyhexanide or octenidine, should be considered.

## Zusammenfassung

Mupirocin ist ein Antibiotikum, das zur Dekolonisierung der nasalen Besiedelung durch Methicillin-resistente *S. aureus* (MRSA) verwendet wird. Eine hochgradige Resistenz wird durch das auf Plasmiden lokalisierte Gen *mupA* verursacht. Eine Mupirocin-Resistenz wurde in Sachsen bisher selten registriert und die Rate der *mupA*-positiven MRSA-Isolate lag an einer Universitätsklinik in Sachsen zwischen 2000 und 2015 bei etwa 1%. 2016/2017 stieg diese Rate jedoch auf fast 20% an. Die Charakterisierung der resistenten Isolate ergab, dass sie einer Variante des Barnimer Epidemiestamms (CC22-MRSA-IV) angehören und außer *mecA* die Resistenzgene *mupA*, *aacA-aphD* und *qacA* sowie meistens *erm*(C) tragen. Daher wird empfohlen, MRSA-Isolate auf Mupirocin-Resistenz zu testen und ggf. genotypisch untersuchen zu lassen. Bei Versuchen der MRSA-Sanierung mit Mupirocin muss zunehmend mit ausbleibenden Erfolgen gerechnet werden. Daher sollte die Dekolonisierung mit anderen Substanzen (Betaisodona, Polihexanid, Octenidin) in Betracht gezogen oder nur bei nachgewiesen sensiblen Isolaten die Dekolonisation mit Mupirocin durchgeführt werden.

## 1 Introduction

Mupirocin is an antibacterial agent that is bactericidal at high concentrations against *Staphylococcus aureus* by irreversibly binding to isoleucyl t-RNA synthetase during ribosomal protein biosynthesis. Mupirocin is principally used for nasal decolonization of methicillin-resistant *S. aureus* (MRSA) [1]. It is also occasionally used for the topical treatment of *S. aureus* skin and soft tissue infections (e.g., impetigo). Low-level mupirocin resistance among *S. aureus* strains (i.e., mupirocin minimum inhibitory concentrations [MICs] between 8 and 256 mg/L as defined by EUCAST) can be conferred by mutations in the isoleucyl-tRNA synthase gene *ileS* [2]. Such strains can still be eradicated by mupirocin treatment, although failures have been observed in some cases. High-level mupirocin resistance (mupirocin MICs >256 mg/L; EUCAST) is usually encoded by *mupA* (also known as *ileS2* or *mupR*, GenBank accession number X75439), a gene that encodes an alternative isoleucyl-tRNA synthase and is predominantly located on conjugative plasmids [2], [3], [4]. 

High rates of high-level mupirocin resistance or of the presence of *mupA* have been observed in some regions, including a rate of 31% among pediatric *S. aureus* isolates in New York City [5], 11% among *S. aureus* isolates in New Zealand [6] and 7 to 24% among MRSA from Trinidad and Tobago [7], [8]. 

In contrast, the rate of high-level mupirocin resistance among MRSA in Germany has been low for many years. A study from Saxony between 2000 and 2011 reported that *mupA* was only detected in 0.64% of clinical MRSA isolates [9]. However, during 2016/2017, the rate of high-level mupirocin resistance in the same hospital where the earlier study [9] was performed increased to nearly 20% among clinical MRSA isolates, and numerous cases were also observed in collaborating healthcare facilities. This dramatic rise in the rate of high-level mupirocin resistance prompted the present investigation. 

## 2 Materials and methods

MRSA isolates were recovered from routine diagnostic and screening samples at Dresden University Hospital (UHD) as well as from another healthcare facility nearby. Additionally, co-operating healthcare facilities submitted isolates for confirmational tests, genotyping and outbreak investigations. These facilities are not named here for reasons of confidentiality.

Susceptibility tests were performed using a commercial, automated microdilution system (VITEK-2, BioMérieux, Nuertlingen, Germany) with Gram-positive susceptibility cards AST-P632, or (prior to May 2017) AST-P619. Mupirocin resistance based on EUCAST breakpoints was determined by gradient diffusion tests (Liofilchem, Roseto degli Abruzzi, Italy; catalogue number 920380), or using VITEK AST-P632 cards. 

Isolates from the UHD were selected for comprehensive characterization by DNA microarray profiling [10], [11] if they originated from outbreak investigations, unusual clinical presentations, diabetological and surgical departments, or intensive care units [9]. Thus, genotyping data were available for one-third to one-half of all MRSA identified during each year of the study period since 2000. Additionally, high-level mupirocin-resistant isolates from the UHD and cooperating healthcare facilities were genotyped as well as high-level mupirocin-resistant CC22-MRSA-IV from Ireland, which were examined using microarray profiling for comparative purposes.

Genotyping by microarray profiling allowed the detection of a wide range of genes associated with virulence or antimicrobial resistance, including *mupA*, as well as the assignment of the isolate to multilocus sequence type (MLST) clonal complexes (CCs), epidemic strains, and staphylococcal cassette chromosome *mec* (SCC*mec*) types. Representative isolates were additionally tested with a second microarray that facilitated SCC*mec* subtyping [12].

## 3 Results

### 3.1 Epidemiology

**Figure 1:**
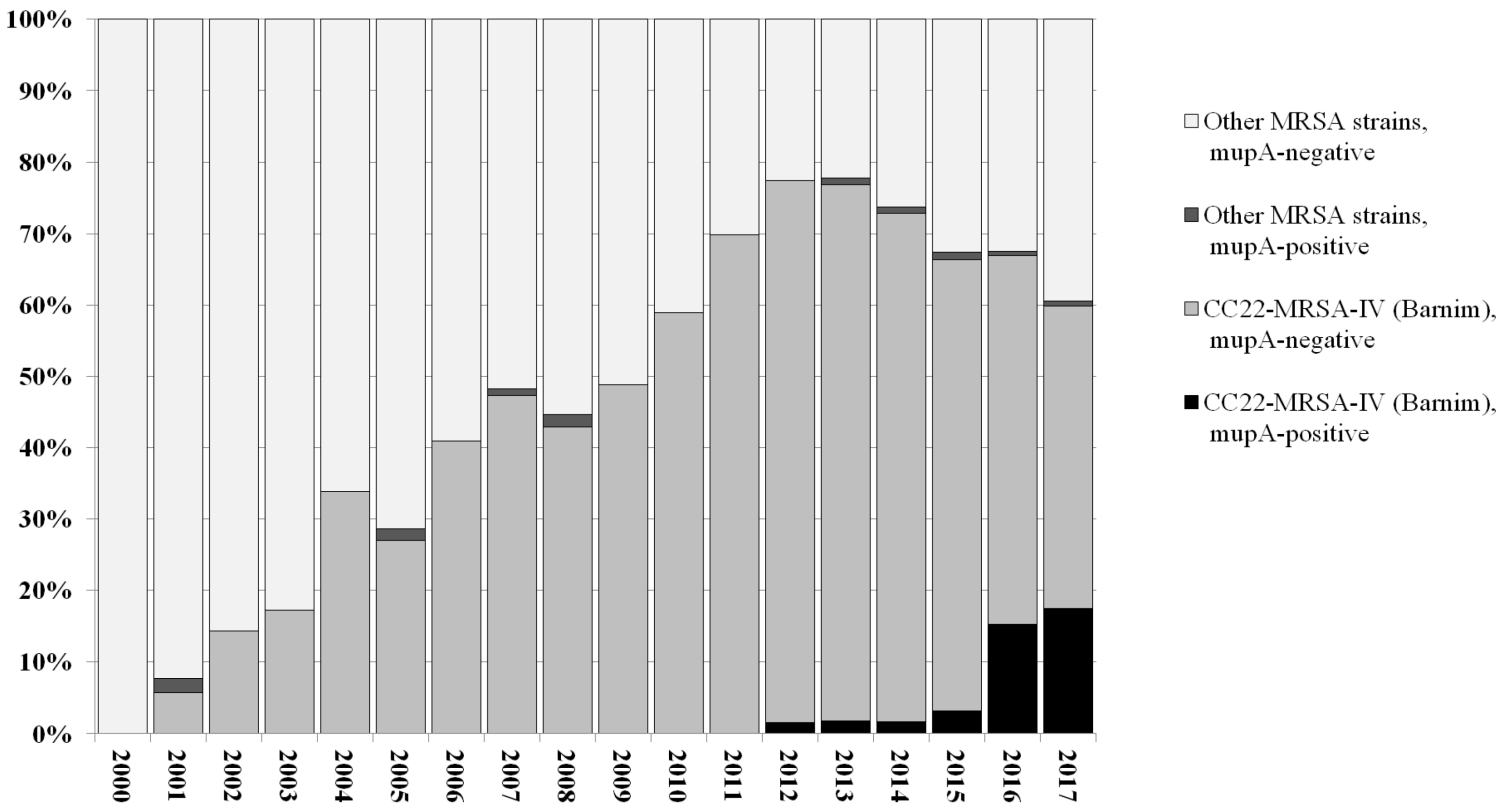
Relative prevalence of *mupA*-positive and -negative MRSA strains, based on genotyping of 1,531 MRSA isolates from UHD, 1.1.2000 to 15.8.2017

In 2016/2017, a steep rise in the prevalence of *mupA*-positive/high-level mupirocin-resistant MRSA recovered at the UHD was observed (Figure 1 (Fig. 1)). The prevalence of *mupA*-positive MRSA increased from 1.1% (mean value for 2000–2015, with an average of 78 isolates genotyped per year) to 15.9% (in 2016, with 151 isolates genotyped) and 17.6% (in 2017, with 125 isolates genotyped by the end of July). While phenotypically determined rates for high-level mupirocin resistance rose in parallel, no clear trend for phenotypic low-level mupirocin resistance was observed. It was detected in approximately 15–25% of routinely tested MRSA isolates.

A total of 1,531 MRSA isolates recovered at the UHD between January 1, 2000 and August 15, 2017 were genotyped by microarray profiling (Figure 1 (Fig. 1)). Sixty-three of these isolates were found to harbor *mupA*. All five *mupA*-positive isolates detected between 2001 and 2008 were assigned to CC45-MRSA-IV, “Berlin Epidemic Strain”. Eleven *mupA*-positive MRSA isolates were identified among isolates recovered between 2012 and 2015, including one CC45-MRSA-IV, two CC1-MRSA-IV and eight CC22-MRSA-IV. Forty-seven *mupA*-positive MRSA were identified among isolates recovered in 2016 and 2017. A single isolate belonged to CC5-MRSA-II (“Rhine-Hesse Epidemic Strain/New York-Japan Clone”) while the remaining 46 were assigned to CC22-MRSA-IV, i.e., to the “Barnim Epidemic Strain”.

An additional 43 *mupA*-positive CC22-MRSA-IV isolates were identified among MRSA isolates from other local healthcare facilities between 2012 and 2017. Since no systematic testing was performed for these facilities, no quantitative prevalence data can be provided, but observations indicate a trend similar to that described above for the UHD. Eight isolates of the high-level mupirocin-resistant CC22-MRSA-IV strain described here were found in these facilities as early 2012/2013, seven were identified in 2014/2015, and 28 in 2016/2017.

### 3.2 Description of the strain

The current outbreak strain was assigned to CC22-MRSA-IV, colloquially known as “Barnim Epidemic Strain” or “UK-EMRSA-15”. 

All characterized isolates (n=97) carried *mecA* (methicillin resistance), *blaZ* (beta-lactamase) and *mupA*. In addition, all isolates were resistant to fluoroquinolones. 

Nearly all isolates harbored *aacA-aphD* (in 95 isolates, 97.9%; confers gentamicin, kanamycin and tobramycin resistance) and *qacC* (in 93 isolates, 95.9%; confers resistance to quaternary ammonium compounds. The majority of isolates (n=73; 75.3%) also carried *erm*(C), resulting in macrolide resistance and constitutive or inducible clindamycin resistance. In one isolate (1.0%) each, the additional resistance genes *tet*(M) (tetracycline resistance) or *qacA* (resistance to quaternary ammonium compounds) were identified. Interestingly, this *qacA*-positive isolate was one of the four *qacC*-negative isolates. 

A single isolate (1.0%) was identified that harbored both *fexA* (florfenicol and chloramphenicol resistance) and *cfr* (resistance to phenicols, lincosamides, oxazolidinones, pleuromutilins, and streptogramin A compounds). 

Four representative CC22-MRSA-IV isolates were SCC*mec* subtyped, and yielded SCC*mec* IVh/j elements that matched the predicted pattern for SCC*mec* IVh/j from the *S. aureus* EMRSA-15 reference strain HO 5096 0412 (GenBank accession number HE681097.1).

None of the isolates investigated harbored genes encoding Panton-Valentine leukocidin (*lukF/S*-PV), the ACME cluster, or the toxic shock syndrome toxin (*tst1*). Enterotoxin genes *sec* and *sel* were observed sporadically, only in two isolates (2.1%).

### 3.3 Comparison to mupA-positive CC22-MRSA-IV from Ireland

Fifty-six *mupA*-positive CC22-MRSA-IV isolates recovered from patients and environmental sites in Irish hospitals between 2004 and 2009, where mupirocin resistance has been a problem for years [13], were investigated for comparative purposes. Microarray genotyping revealed the presence of an ACME II element in 14/56 (15%) of these isolates, which is consistent with the findings of a previous study from Ireland [14]. The enterotoxin genes *sec*/*sel* were more common than in the isolates from Saxony (34 isolates; 60.7%). Regarding resistance genes, *erm*(C) was present in 52 (92.9%), *lnu*(A) in 23 (41.1%), *aacA-aphD* in 46 (82.14%), *aadD* in 20 (35.7%), and *qacC* in 3 isolates (5.4%). 

The latter three isolates (recovered in 2006 and 2007) most closely matched the Saxon outbreak strain, being positive for *erm*(C), *aacA-aphD* and *qacC*, but negative for ACME II and *sec*/*sel*. However, they differed in the presence of *lnu*(A), *aadD*, and (in two out of three) *cat*. 

## 4 Discussion

CC22-MRSA-IV, “Barnim Epidemic Strain” or “UK-EMRSA-15” has been present in Germany since 1996 [15] and in Dresden since 2001 [9]. It became increasingly abundant during the following decade, and in some years, nearly 80% of MRSA isolates were assigned to this strain [9], [12]. This strain cannot only be found in hospitals, but also in nursing homes, care facilities etc., and they can be transmitted to the community. Because of its epidemiological relevance, any changes in its genetic content may be important. During the last 20 months, we have observed a steep rise of *mupA*-positive MRSA at UHD as well as at other, cooperating healthcare facilities in south-eastern Saxony. The present study has revealed that this trend can be attributed to the increasing presence of a particular variant of CC22-MRSA-IV, “Barnim Epidemic Strain”, which carries a unique combination of *mupA*, *aacA-aphD*, and *qacA*. 

We compared the current outbreak strain to isolates from Ireland, where mupirocin-resistant CC22-MRSA-IV has been a problem for years. There were no identical isolates in a collection of 56 *mupA*-positive CC22-MRSA-IV Irish isolates investigated, and thus there was no evidence for direct importation of isolates from Ireland to Saxony. However, three Irish isolates proved to be similar, although they harbored additional resistance markers not found in the Saxon outbreak strain. This warrants further studies on the *mupA*-encoding plasmids present in Irish and German isolates, and possibly a broader screening for matching Irish isolates. However, this is beyond the scope of the present outbreak investigation. An epidemiological link to Ireland, such as travel histories of patients or staff members, has not yet been established in retrospect. This might be rather complicated, given that the first cases appeared five years ago, some of the patients in question have conditions that make it impossible to discuss previous travel histories, and also due to privacy concerns. 

Based on these observations, we recommend monitoring for the possible presence of high-level mupirocin-resistant MRSA and genotyping of suspect isolates, not only in the State of Saxony but also in adjacent regions and in patients with a recent history of travel to or hospitalization in Saxony. Additionally, in order to prevent long-distance spread of MRSA and other multidrug-resistance (MDR) organisms, travel histories should be obtained for all patients, not only for patients with suspected travel-associated disease. If travel-associated MDR organisms are detected, they should generally be preserved and submitted for molecular typing. 

Furthermore, we recommend more cautious use of mupirocin. This substance should be reserved only for MRSA decolonization. It should not be generally used as a topical treatment for *S. aureus*-associated skin disorders, although replacement by other substances such as fusidic acid might also result in the emergence of resistance [6]. As for other antibiotics, the use of mupirocin should be guided by susceptibility tests. In cases of proved resistance, or in regions with a high prevalence of *mupA*-positive MRSA such as, recently, south-eastern Saxony, other substances should be used for MRSA decolonization. Chlorhexidine might not be a suitable alternative because of the presence of the *qacC* gene in the current outbreak strain. Thus, options include betaisodona, polihexanide or octenidine. For the latter, it has been shown on several MRSA strains including “UK-EMRSA-15” that sub-lethal doses do not select for resistance [16].

The current outbreak emphasizes the need for a constant surveillance – both molecular and with regard to resistance phenotypes – that facilitates intervention in case of the spread of epidemic strains that might endanger patients and economically burden the healthcare system. 

## Notes

### Competing interests

The authors declare that they have no competing interests.

### Funding

There was no external funding for this study. 

### Acknowledgements

The authors thank the infection control nurses at UHD and the laboratory staff of the Institute for Medical Microbiology and Hygiene, Technische Universität Dresden.
